# Diagnosis and Surgical Treatment of Thoracic Dorsal Arachnoid Web: A Report of Two Cases

**DOI:** 10.1155/2020/8816598

**Published:** 2020-09-12

**Authors:** Junichi Inoue, Naohisa Miyakoshi, Michio Hongo, Takashi Kobayashi, Toshiki Abe, Kazuma Kikuchi, Eiji Abe, Yuji Kasukawa, Yoshinori Ishikawa, Daisuke Kudo, Hayato Kinoshita, Ryota Kimura, Yoichi Shimada

**Affiliations:** ^1^Department of Orthopedic Surgery, Akita University Graduate School of Medicine, Akita, Japan; ^2^Department of Orthopedic Surgery, Akita Kosei Medical Center, Akita, Japan; ^3^Department of Orthopedic Surgery, Omagari Kosei Medical Center, Akita, Japan

## Abstract

**Introduction:**

An arachnoid web (AW) is a relatively rare disease and shows clinical symptoms and radiological findings similar to those of an arachnoid cyst (AC) or spinal cord herniation (SCH). Since the operative procedures for an AW are generally different from those intrathecal disorders, correct preoperative differential diagnosis is important. The purposes of this study were to report the usefulness of magnetic resonance imaging (MRI) and computed tomography (CT) myelography for diagnosing AW and to show the histological findings and clinical results. *Case Description.* Two patients, a 79-year-old man and a 43-year-old woman, are presented. The primary diagnoses were AC with ossification of the ligamentum flavum and epidural hematoma, respectively, in previous hospitals. They were finally diagnosed by the characteristic MRI and CT myelogram finding called the “scalpel sign.” Histological findings showed epithelial cells and fibrous tissue derived from arachnoid tissues and microcalcifications. After surgery, the scalpel sign has vanished, and aggravation of their symptoms was prevented.

**Conclusion:**

An AW is refractory, but early detection by MRI and CT myelography and early treatment improve outcomes after surgery.

## 1. Introduction

An arachnoid web (AW) is relatively rare and shows clinical symptoms and radiological findings similar to an arachnoid cyst or spinal cord herniation. Spinal arachnoid cysts are cerebrospinal fluid (CSF) pockets contained by the arachnoid mater, and intradural onset occasionally causes spinal cord compression. An AW has been reported as a variant of arachnoid cyst [1]. The webs represent intradural extramedullary transverse bands of arachnoid membrane, generally located at the dorsal side of the spinal cord. Although the mechanisms of AWs have been explained with quantitative measurement on magnetic resonance imaging (MRI) [1], histological findings have rarely been reported. The details of two AW cases treated surgically, including their histological findings, are presented.

## 2. Case Presentations

### 2.1. Case 1

#### 2.1.1. History and Examination

A 79-year-old man presented with frequent falls and an unsteady gait. He had a three-year history of recurrent falls, and a primary diagnosis of arachnoid cyst and ossification of the ligamentum flavum was made at another orthopedic clinic. His past history included asthma, gastric ulcer, spontaneous pneumothorax, prostatic cancer, dementia, hypertension, hyperlipidemia, paroxysmal atrial fibrillation, and herpes zoster. His symptoms worsened gradually, and he was referred to our hospital. On neurological examination, he could not stand on his left foot. Manual muscle testing showed no muscle weakness. There was no numbness or sensory disturbance of the upper and lower extremities and trunk. Deep tendon reflexes, the patellar tendon and Achilles tendon reflexes, showed hyporeflexia.

**Figure 1:**
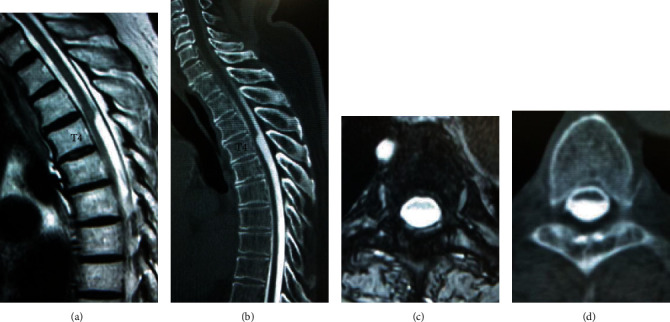
Sagittal MRI and CT myelography demonstrate a dorsal indentation, the so-called “scalpel sign,” at the T4 level.

Spinal cord deviation at the T4 level was seen on MRI. T2-weighted sagittal MRI demonstrated a sharp dorsal indentation of the spinal cord (Figures 1(a) and 1(c)). CT myelography showed the same changes of the spinal cord as MRI (Figures 1(b) and 1(d)). This was diagnosed as an arachnoid cyst possibly consistent with AW based on the findings of MRI and CT myelography. The compression of the spinal cord was considered to have caused his gait disturbance, and surgery was scheduled 6 months after his first visit.

#### 2.1.2. Operation

**Figure 2:**
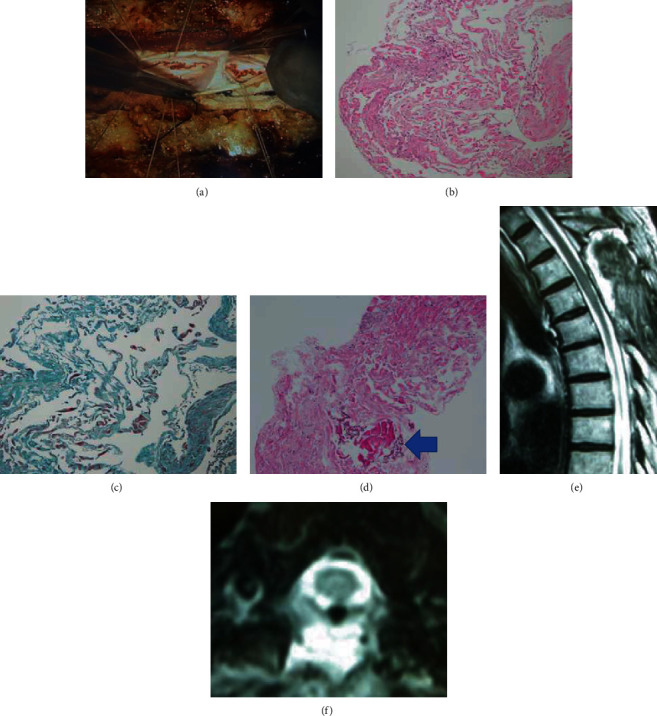
(a) Intraoperative findings show a membrane-like structure in the subarachnoid space. (b) Fibrous tissue seen on hematoxylin and eosin staining. (c) Epithelial membrane antigen-positive epithelial tissue is seen. (d) Calcification seen on hematoxylin and eosin staining (arrow). (e, f) On postoperative MRI, the spinal cord deviation and scalpel sign have disappeared.

After laminectomy and durotomy at T2, T3, and T4, a conglutinated arachnoid membrane covering the spinal cord and indentation of the spinal cord were observed. Intraoperatively, unidirectional pulsation was recognized from the head side to the caudal side. The arachnoid membrane was then resected (Figure 2(a)). Histological findings of the resected specimen included epithelial cells, indicating that it was derived from arachnoid tissue. No inflammatory cells or neoplastic lesions were detected (Figures 2(b)–2(d)).

#### 2.1.3. Postoperative Course

After the operation, the patient was gradually able to walk more smoothly and stably and fall less frequently than before. Seven weeks later, he was discharged from the hospital ambulatory. His postoperative MRI showed that the scalpel sign had disappeared, and the spinal cord was shifted posteriorly (Figures 2(e) and 2(f)).

### 2.2. Case 2

#### 2.2.1. History and Examination

A 43-year-old woman presented with a 1-month history of back pain. A primary diagnosis of thoracic epidural hematoma was made at another general hospital. Her history included untreated diabetes. Her back pain gradually worsened, and she was referred to our hospital. On neurological examination, manual muscle testing showed no muscle weakness. There was no numbness or sensory disturbance of the upper and lower extremities and trunk. Deep tendon reflexes, the patellar tendon and Achilles tendon reflexes, showed hyporeflexia.

**Figure 3:**
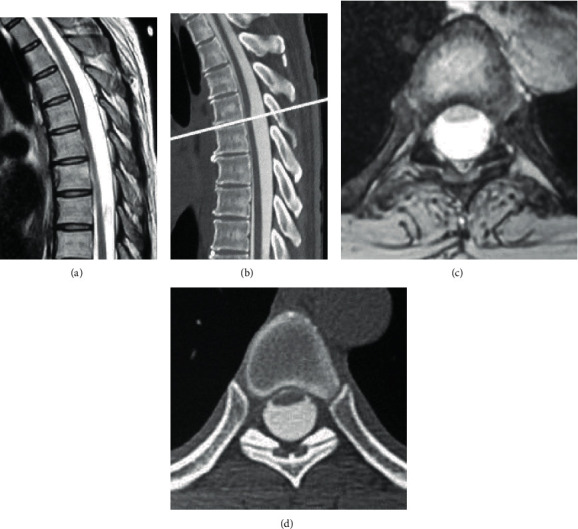
Preoperative T2-weighted MRI and CT myelography. (a, b) Sagittal MRI and CT myelography show the “scalpel sign.” The white line indicates the T5/T6 disc level. (c, d) Axial MRI and CT myelography show spinal cord deviation.

Spinal cord deviation at the T5 level was seen on MRI. T2-weighted sagittal MRI demonstrated a sharp dorsal indentation of the spinal cord (Figures 3(a) and 3(c)), and CT myelography showed the same change of the spinal cord (Figures 3(b) and 3(d)). This was diagnosed as an arachnoid cyst possibly consistent with AW based on the MRI and CT myelography findings. The compression of the spinal cord was thought to have caused her back pain. She was first treated for refractory diabetes, and then, surgery was performed 2 months later. During this period, she developed anterior chest and bilateral low limb pain.

#### 2.2.2. Operation

**Figure 4:**
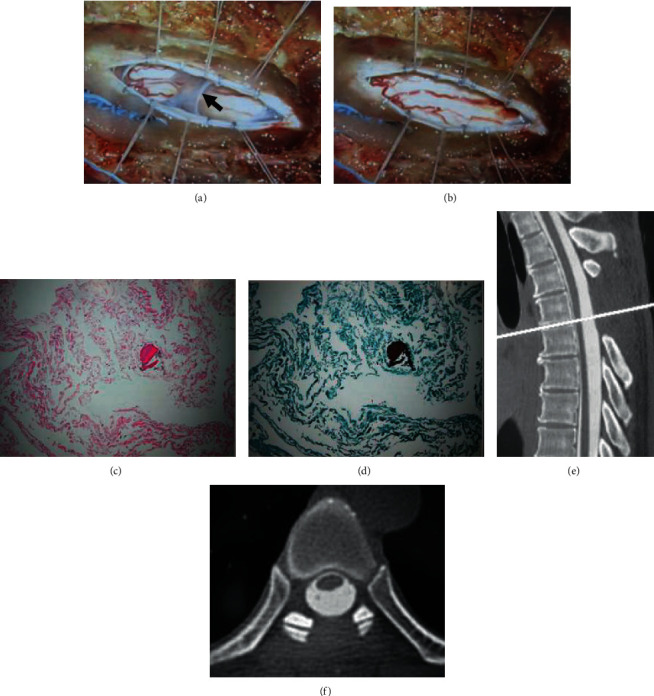
(a) The right side is cranial, and the left side is caudal. The dura and arachnoid are incised. The arachnoid web is still present as a restiform structure (black arrow). (b) After resection of the arachnoid web. (c) Hematoxylin and eosin staining shows fibrous tissue and microcalcifications. (d) Elastica-Masson staining shows epithelial tissue. (e, f) Postoperative sagittal and axial CT myelography show no spinal cord deviation, and the “scalpel sign” has vanished. The white line shows the T5/T6 disc level.

After laminectomy and durotomy at T4, T5, and T6, a conglutinated arachnoid membrane covering the spinal cord and indentation of the spinal cord were observed. Intraoperatively, unidirectional pulsation was recognized from the head side to the caudal side. The arachnoid membrane was then resected (Figures 4(a) and 4(b)). Histological findings of the resected specimen included fibrous tissue and microcalcifications consistent with AW. No inflammatory cells or neoplastic lesions were detected (Figures 4(c) and 4(d)).

#### 2.2.3. Postoperative Course

After surgery, there was no aggravation of her symptoms, but there was no improvement. The scalpel sign on MRI seen before surgery has vanished (Figures 4(e) and 4(f)).

## 3. Discussion

AW is a rare disease with only 61 reports so far in the literature [1–8]. Since surgery is the only curative treatment, the correct diagnosis is very important. Randall et al. stated that differentiating AW and SCH was of vital importance from a surgical perspective. While the surgical access for these lesions is similar, definitive treatment of SCH requires division of the dentate ligaments such that the spinal cord can be rotated for inspection of the ventral cord and dura, whereas AWs are treated with lysis of the web without the need for such exposure or repair [9]. Generally, differential diagnosis from arachnoid cyst or spinal cord herniation is necessary.

### 3.1. Imaging Diagnosis

Imaging findings are important in diagnosing AW. Several characteristic imaging findings such as the scalpel sign, syringomyelia, and dorsal indentation (ventral deviation of the spinal cord) have been reported on MRI and CT myelography [3–10]. The scalpel sign is also called the “scalpel blade” sign, because the shape of the spinal cord in the sagittal section of MRI resembles a scalpel blade [3, 7]. An AW impedes CSF flow and dilates the subarachnoid space, suggesting a spinal cord deviation (spinal cord dorsal indentation). This is thought to involve an AW's “one-way valve mechanism” [2]. The membrane structure of the AW can create unidirectional flow in the spinal fluid, which causes the AW to compress the spinal cord.

The findings of syringomyelia are also characteristic imaging findings of AW [3–10]. Spinal cavities may be found at the upper and lower levels of an AW, and the mechanism has been reported to be due to CSF pressure differences. In the present two cases, syringomyelia was not found. Chellathurai et al. examined the pathophysiology of the ventral displacement of the dorsal spinal cord between D3 and D7 using MRI correlation and severity grading [10]. Schultz et al. showed that AW and spinal cord herniation can be reliably distinguished on imaging by scrutinizing the nature of the dorsal indentation and the integrity of the ventral subarachnoid space at the level of the cord deformity [9].

### 3.2. Pathological Findings

Previous reports have suggested that an AW is a subtype of arachnoid cyst or a change in the arachnoid after infection or trauma [7], but no definitive conclusion has been made. Chang et al. stated that the histopathology of AW showed connective tissue, small numbers of CD3+ T cells, and an asymptomatic small ossification [2]. The ossification of the ligamentum flavum associated with arachnoiditis and the presence of CD3-positive cells suggests the possibility of an inflammatory process. They suggested that inflammation in the epidural space may have extended into the subarachnoid space, thereby leading to the formation of the relatively thickened arachnoid membrane. In the present cases, the arachnoid tissue and epithelial tissue were confirmed. No inflammatory cells were detected, but calcifications were detected. Thus, the present cases might have been associated with arachnoiditis.

### 3.3. Treatment

No dramatic improvements were observed after AW resection in the present cases, but surgery is necessary to prevent the progression of symptoms. As for the treatment of AW, all cases reported in the literature were treated surgically. The surgical treatment is removal of the AW after dural incision, with no fixation or decompression [6].

Several articles have reported that AW patients' symptoms improved after surgery. Nisson et al. reported that, after surgery, 91% of patients showed neurological improvement [8]. On the other hand, Hirai et al. showed that the postoperative symptoms tended to improve in 5 cases, but numbness of the lower limbs remained in 3 cases, and bladder disorders did not change after operation in 1 case [6]. At this time, there are no clear reports on the risks of postoperative residual symptoms.

In the present cases, it is probable that postoperative symptoms did not improve significantly because of the long waiting period between the onset of symptoms and surgery and the uncontrolled diabetes, which could cause numbness. Identification of postoperative prognostic factors requires further study.

In case 1, the symptoms recovered slightly after the operation. However, the patient fell over after discharge and suffered a vertebral fracture. In case 2, her symptoms did not improve, and she continues to take medications, mainly analgesics. The neurologist in our hospital suggested that the remaining pain could be the result of atypical diabetic neuropathy. Lozeron et al. showed that atypical diabetic neuropathy disease has several patterns and sometimes affects the thoracolumbar region. In fact, the patient's glycemic control has been poor, so the endocrinologist still continues a strict regimen of treatment [11].

### 3.4. CSF Dynamics

Chang et al. measured the flow rate of CSF using MRI in a cardiac-gated phase-contrast cine-mode to clarify the one-way valve-like function of an AW [2]. In the present cases, the CSF velocity from the dorsal head to the caudal direction was slow, whereas the CSF velocity from the caudal to the cranial direction was not reduced. They also showed that CSF speed improved after AW resection. These results show that an AW has a one-way function and impedes CSF flow and that excision improves CSF flow. Such MRI imaging is considered to be a useful method that contributes to elucidation of the pathological condition of AW.

## 4. Conclusion

An AW can be diagnosed by characteristic MRI findings in the early stage. Though symptom improvement is difficult to achieve even with surgery for AW, surgery is useful to prevent aggravation of symptoms, and earlier surgery could improve treatment outcomes.

## Data Availability

Informed consent was obtained from all of the patients in the present study.
